# ATP mediates the interaction between human blastocyst and endometrium

**DOI:** 10.1111/cpr.12737

**Published:** 2019-12-10

**Authors:** Xiao‐Wei Gu, Yan Yang, Tao Li, Zi‐Cong Chen, Tao Fu, Ji‐Min Pan, Jian‐Ping Ou, Zeng‐Ming Yang

**Affiliations:** ^1^ College of Veterinary Medicine South China Agricultural University Guangzhou China; ^2^ Center for Reproductive Medicine The Third Affiliated Hospital of Sun Yat‐Sen University Guangzhou China

## Abstract

**Objective:**

Embryo implantation needs a reciprocal interaction between competent embryo and receptive endometrium. Adenosine triphosphate (ATP) produced by stressed or injured cells acts as an important signalling molecule. This study aims to investigate whether adenosine triphosphate (ATP) plays an important role in the dialogue of human blastocyst‐endometrium.

**Materials and methods:**

The concentration of lactate was analysed in culture medium from human embryos collected from in vitro fertilization patients. Extracellular ATP was measured by ATP Bioluminescent Assay Kit. Ishikawa cells and T‐HESCs were treated with ATP, ATP receptor antagonist, ATP hydrolysis enzyme or inhibitors of ATP metabolic enzymes. The levels of gene expression were evaluated by real‐time PCR and immunoassay.

**Results:**

We showed that injured human endometrial epithelial cells could rapidly release ATP into the extracellular environment as an important signalling molecule. In addition, blastocyst‐derived lactate induces the release of non‐lytic ATP from human endometrial receptive epithelial cells via connexins. Extracellular ATP stimulates the secretion of IL8 from epithelial cells to promote the process of in vitro decidualization. Extracellular ATP could also directly promote the decidualization of human endometrial stromal cells via P2Y‐purinoceptors. More importantly, the supernatants of injured epithelial cells clearly induce the decidualization of stromal cells in time‐dependent manner.

**Conclusion:**

Our results suggest that ATP should play an important role in human blastocyst‐endometrium dialogue for the initiation of decidualization.

## 1 INTRODUCTION

Successful embryo implantation involves a sophisticated molecular interaction between the competent embryo and receptive endometrium.^1^ Once embryo implantation occurs, the structural and molecular changes of endometrial luminal epithelial cells are followed by decidualization of the endometrial stromal cells in rodents and primates.^2^ Decidualization of the human endometrium is a complex and dynamic process involving a dramatic morphological and functional transformation of human endometrial stromal cells.^3^ Impaired embryo implantation and decidualization results in pregnancy loss and infertility and may be the main cause of the lower implantation rate in assistive reproductive technology.^4^, ^5^ Given the pervasiveness of this problem, many efforts have been made to study the receptivity of the endometrium for the blastocyst.^6^, ^7^ However, the molecular mechanism underlying the crosstalk between the embryo and endometrium remains unclear.

The concentration of ATP in the extracellular space is very low (nmol/L) in resting cells and healthy tissues. However, the intracellular ATP concentration generated by glycolysis and oxidative phosphorylation by mitochondria can reach 1‐10 mmol/L.^8^ Extracellular ATP, a signalling molecule, is an important nonprotein element of “damage‐associated molecular pattern” molecules (DAMPs) that are released from stressed, injured or dying cells. The local concentration of extracellular ATP can reach mmol/L level in released site in vivo.^9^ In addition, ATP is released via several non‐lytic mechanisms, including exocytosis, microvesicles and transmembrane channels.^10^ The connexin and pannexin hemichannels are the main transmembrane channels for ATP release and have been detected in multiple human tissues under both physiological and pathological conditions.^11^ Most studies have shown that connexin 43 (Cx43), connexin 26 (Cx26) and pannexin1 (Panx1) frequently regulate ATP release in different cell types.^12^ Once released, extracellular ATP is rapidly hydrolysed into ADP and AMP by ectonucleoside triphosphate diphosphohydrolase 1 (CD39), and AMP is further hydrolysed into adenosine by ecto‐5′‐nucleotidase (CD73).^13^ Released ATP can activate a series of signalling pathways via its specific receptor. Extracellular ATP receptors are known as P2 receptors, which include the P2X (P2XRs) and P2Y (P2YRs) receptor subfamilies and are distributed in many tissues in mammals. P2XRs are ATP‐gated cationic channels that allow ion exchange. P2YRs are G‐protein–coupled transmembrane receptors that increase the concentration of either intracellular Ca2 + or cyclic adenosine monophosphate (cAMP) 14, 15, 16). ATP is related to a variety of physiological processes, including inflammation, angiogenesis and the wound‐healing response.^17^, ^18^, ^19^ It has been shown that many inflammation‐related genes, such as prostaglandin E2 (PGE2), TNF, IL6 and IL1β, are involved in embryo implantation and decidualization.^20^ However, the potential ATP signalling cascades involved in decidualization remain unexplored.

Although the effects of endometrial injury on in vitro fertilization (IVF) outcomes remain controversial because of differences in methods used to induce injury,^21^ many studies have shown that local endometrial injury in the preceding cycle of ovarian stimulation improves implantation and clinical pregnancy rates in women undergoing IVF.^22^, ^23^, ^24^ However, whether the injured endometrium releases ATP has not been reported.

In the present study, our results showed that blastocyst‐derived lactate might induce ATP release from human endometrial epithelial cells via connexins. We also identified IL8 as the paracrine factor that drives ATP actions on adjacent epithelial cells. Furthermore, extracellular ATP from injured epithelial cells can promote the decidualization of human endometrial stromal cells in vitro.

## 2 MATERIALS AND METHODS

### 2.1 Medium collection from cultured human embryos

All patients underwent IVF treatment according to standard protocols at the Center for Reproductive Medicine, Third Affiliated Hospital of Sun Yat‐sen University in Guangzhou. Briefly, oocytes were incubated with spermatozoa in fertilization medium (G‐IVF™ Plus, Vitrolife) until the presence of two pronuclei and a second polar body was observed. Then, embryos were cultured in 30 μL of pre‐equilibrated cleavage medium (G1™ Plus, Vitrolife) at 37°C in an atmosphere of 6% CO_2_ and 5% O_2_ for 48 hours. On day 3, usable embryos were washed and cultured in 30 μL of pre‐equilibrated blastocyst medium (G2™ Plus, Vitrolife) in an atmosphere of 6% CO_2_ and 5% O_2_ at 37°C. Culture medium without embryos was used as the control. On day 5, blastocysts were scored according to the grading system developed by Gardner using a time‐lapse microscope. Embryos that were scored >3BB were preserved by freezing. Embryos that were scored ≤3BB were cultured further in the previous medium until day 6. After embryos that were scored ≥4BC were frozen on day 6, the remaining culture medium of these embryos and control medium were collected and stored at −80°C for further analysis. All human procedures were approved and conformed to recognized standards by the Institutional Committee on the Use of Human Subjects in Third Affiliated Hospital of Sun Yat‐Sen University.

### 2.2 Cell lines

T‐HESCs (human telomerase reverse transcriptase‐immortalized human endometrial stromal cells) and AN3‐CA cells were purchased from American Type Culture Collection (ATCC). T‐HESCs were cultured in DMEM/F12 medium without phenol red (Sigma) supplemented with 10% charcoal/dextran‐treated foetal bovine serum (cFBS) (Biological Industries), 1.5 g/L sodium bicarbonate, 1% ITS‐G (Gibco), and 500 ng/mL puromycin. Ishikawa cells were also obtained from ATCC. Ishikawa cells were cultured in phenol red‐free DMEM/F12 medium supplemented with sodium pyruvate, penicillin/streptomycin, and 10% foetal bovine serum (FBS) (Biological Industries). AN3‐CA cells were cultured in Eagle's Minimum Essential Medium (Sigma) supplemented with 10% FBS, 2.2 g/L sodium bicarbonate, 0.292 g/L L‐glutamine, and penicillin/streptomycin. All cells were maintained in an incubator at 37°C under a 5% CO2 atmosphere. For further experiments, cultured cells were treated with ATP (sigma), suramin (100 μmol/L, Santa Cruz Biotechnology), apyrase (1 U/mL, Sigma), ARL67156 (200 μmol/L, Sigma), carbenoxolone (Sigma), IL8 recombinant protein (R&D), cathepsin B recombinant proteins (R&D), trypsin (Amerso) and lactate (Aladdin).

### 2.3 Decidualization of human endometrial stromal cells

In vitro decidualization was performed as previously described.^25^ T‐HESCs were seeded in 12‐well plates and cultured in DMEM/F12 supplemented with 10% cFBS and 1% penicillin‐streptomycin until ~80% confluency. To induce decidualization in vitro, cell media was changed to DMEM/F12 with 2% cFBS and 1% penicillin‐streptomycin and cells were treated with 0.1/0.5 mmol/L db‐cAMP (Sigma) and 1 μmol/L medroxyprogesterone acetate (MPA) (Sigma) for 48 hours. Decidualization was evaluated by detecting the expression levels of insulin‐like growth factor binding protein‐1 (IGFBP1), prolactin (PRL) and forkhead box o1 (FOXO1) mRNA.

### 2.4 Real‐time RT‐PCR

Real‐time RT‐PCR was performed as previously described.^26^ Briefly, total RNA from cells was obtained with TRIzol Reagent (Invitrogen™). Genomic DNA was digested with RQ1 deoxyribonuclease I (Promega) to purify RNA. Then, 500 ng of RNA from each sample was reverse transcribed into cDNA with the PrimeScript reverse transcriptase reagent kit (TaKaRa) according to the manufacturer's instructions. For real‐time PCR, cDNA was amplified using a SYBR Premix Ex Taq kit (TaKaRa) following the manufacturer's instructions on the CFX96 Touch™ Real‐Time System (BioRad). The mRNA level of each target gene was normalized to RPL7 mRNA. The relative changes in gene expression from real‐time PCR were calculated using the 2^−△△Ct^ method. The corresponding primer sequences are listed in Table 1.

**Table 1:** Primers used in this study

Gene	ID	Primers (5′‐3′)	Products (bp)
*IGFBP1*	NM_001013029	CCAAACTGCAACAAGAATG GTAGACGCACCAGCAGAG	87
*PRL*	NM_000948	AAGCTGTAGAGATTGAGGAGCAAA TCAGGATGAACCTGGCTGACTA	76
*FOXO1*	NM_002015	CGAGCTGCCAAGAAGAAA TTCGAGGGCGAAATGTAC	105
*PLZF*	NM_006006	TCACATACAGGCGACCACC CTTGAGGCTGAACTTCTTGC	144
*IL8*	NM_000584	AAGGTGCAGTTTTGCCAAGG CAACCCTCTGCACCCAGTTT	204
*MCP1*	NM_002982	AATCAATGCCCCAGTCACCT CTTCTTTGGGACACTTGCTGC	107
*LIF*	NM_001257135	GATGGGCTTCGAGAAAGA CAGGGAGATGAGGTGATGG	94
*IL1β*	NM_000576	AGCTCGCCAGTGAAATGATG CTTGCTGTAGTGGTGGTCGG	161
*Cx26*	NM_004004	GTGGTGGACCTACACAAGCA CATGGAGAAGCCGTCGTACA	94
*Cx43*	NM_000165	CAAAATCGAATGGGGCAGGC CAAAATCGAATGGGGCAGGC	136
*PANX1*	NM_015368	GGCTGCATAAGTTTTTCCCCT TTGCAGCCTTAATTGCACGG	162
*RPL7*	NM_000971	CTGCTGTGCCAGAAACCCTT TCTTGCCATCCTCGCCAT	194
*P2X4*	NM_002560	GTCATCGGGTGGGTGTTTGT TGGTCGCATCTGGAATCTCG	244
*P2X6*	NM_002560	ACTGCCGCTATGAACCACAA CTGATGCCTACAGAGCCACC	121
*P2Y1*	NM_002563	CCGTCTCCTCGTCGTTCAAA ACGTACAAGAAGTCGGCCAG	199
*P2Y14*	NM_001081455	AGCTGAACGTGTTTGTGTGC GGAACAGCAAGGAGGAGCA	202

### 2.5 Western blot analysis

Western blotting was performed as previously described.^27^ In brief, cultured cells were lysed with RIPA buffer containing protease inhibitors (Roche). Protein concentrations were quantified using the BCA protein assay kit (Pierce). Isolated protein lysates were run on a 10% SDS‐PAGE gel and transferred onto polyvinylidene difluoride (PVDF) membranes (Millipore). After blocking with 5% nonfat milk in TBS‐T at room temperature, PVDF membranes were incubated overnight at 4°C with the corresponding antibodies, including phospho‐Stat3 (#9145, 1:1000, Cell Signaling), Foxo1 (#2880, 1:1000, Cell Signaling) or β‐Actin (#4970, 1:1000, Cell Signaling). Then, the membranes were incubated with corresponding HRP‐conjugated secondary antibodies (1:5000) in 5% nonfat milk in TBS‐T. Signals were visualized with an ECL kit (Millipore).

### 2.6 Extracellular ATP assay

Cells grown in 35‐mm dishes were cultured until confluence in growth media and starved with basal medium overnight. Then, the cells were extensively wounded with 200 μL tips and multiple linear scratches. The culture medium from scratched cells or lactate‐treated cells was collected at different time points. The cell supernatant was centrifuged at 1000 g for 5 minutes to remove cell debris. ATP in the supernatant was measured using an ATP Bioluminescent Assay Kit following the manufacturer's instructions (Sigma). Briefly, 100 μL of supernatant was mixed with 100 μL of ATP Assay Mix solution diluted 50 times with dilution buffer, and luminescence was quantified on a GloMax Fluorometer (Promega). The luminescence values of ATP standards were determined in a similar manner. ATP concentration was calculated according to an ATP‐standard curve.

### 2.7 Lactate assay

The concentration of lactate in the collected medium from cultured human embryos was measured using a L‐lactate assay kit (Cayman) according to the manufacturer's instructions. The kit has a detection sensitivity limit of 25 µmol/L L‐lactate. The assay was performed using a fluorescence spectrophotometer at an excitation wavelength of 530‐540 nm and an emission wavelength of 585‐595 nm.

### 2.8 Human IL8 measurement by ELISA

Ishikawa cells in 24‐well culture plates were starved overnight with DMEM/F12 medium without FBS and treated with ATP for 3, 6, 12 and 24 hours. The culture medium was collected and centrifuged at 1000 g for 5 minutes to remove precipitated cells. The levels of human IL8 in the culture medium were determined using commercial ELISA kits (BioLegend) according to the manufacturer's instructions. The level of human IL8 measured was normalized to the level of total protein in the cultured cells for each condition using a BCA kit.

### 2.9 Cell viability assay

Cell viability was measured using the Cell Counting Kit‐8 (Sigma) according to the manufacturer's instructions. Briefly, T‐HESCs were seeded in 96‐well plates and incubated with CCK‐8 for 4 hours. The spectrometric absorbance of each well was recorded at 450 nm using a 96‐well plate reader (Biotech). The level of cell viability was calculated as the OD value of the sample/average OD value of the control × 100%.

### 2.10 Statistical analyses

All the treatments were performed in triplicate, and at least three independent experiments were carried out. Values are presented as the mean ± standard deviation (SD) unless stated otherwise and analysed using GraphPad Prism 5 software. Differences between groups were assessed by paired t test. A value of *P* < .05 was considered statistically significant.

## 3 RESULTS

### 3.1 ATP release from injured epithelial cells

**Figure 1:**
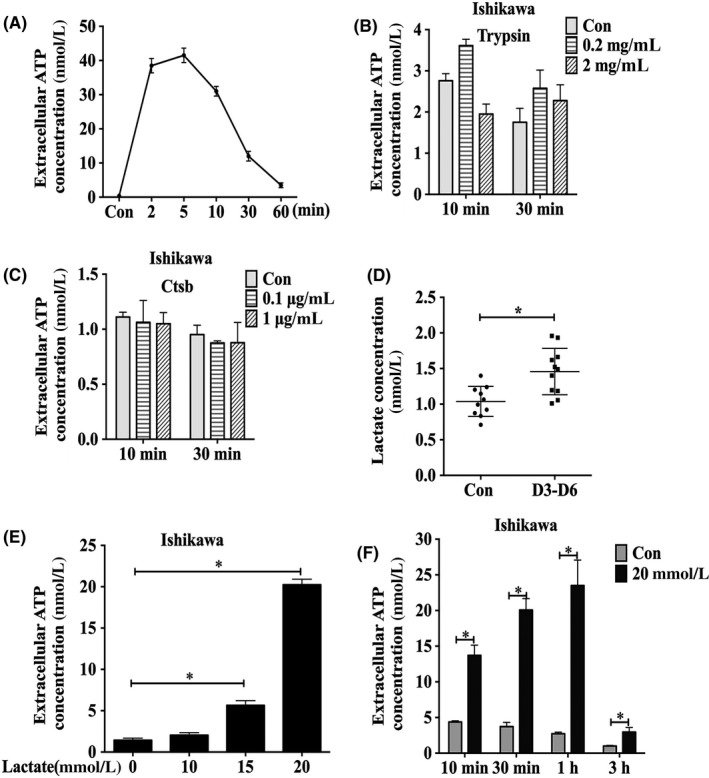
ATP release from Ishikawa cells. A, The ATP levels in the cell medium of scratched Ishikawa cells were measured by ATP Bioluminescent Assay Kit at different time points. B, Levels of extracellular ATP released when Ishikawa cells were treated with different concentrations of trypsin at different time points. C, Levels of extracellular ATP released when Ishikawa cells were treated with different concentrations of Ctsb at different time points. D, Lactate level in human embryo culture medium from days 3 to 6 in vitro was assayed by L‐lactate assay kit. E, Levels of extracellular ATP released when Ishikawa cells were treated by different concentrations of lactate for 1 h. F, Levels of extracellular ATP released when Ishikawa cells were treated with 20 mmol/L lactate at different time points. Data are presented as the mean ± SD, *P < .05

Ishikawa cells, a human endometrial epithelial cell line, were mechanically scratched with pipette tips. The concentrations of ATP in cell medium were examined at 2, 5, 10, 30 and 60 minutes after the Ishikawa cell monolayer was scratched. Compared with those of the nonscratched cell monolayer, the extracellular ATP levels at 2, 5 and 10 minutes after scratching were dramatically increased. However, the ATP level was obviously decreased at 30 minutes and dropped almost to background levels at 60 minutes (Figure 1A). This may be related to the rapid degradation of ATP by ectonucleotidases in the extracellular environment. This result suggested that injured Ishikawa cells could release ATP.

### 3.2 Lactate induction of ATP release from epithelial cells

Our data showed that ATP could be released from injured epithelial cells. We asked how ATP release was stimulated under physiological conditions. A previous study demonstrated that embryo‐derived trypsin was able to activate the epithelial Na^+^ channel to trigger prostaglandin E_2_ release.^28^ We hypothesized that embryonic trypsin might induce the release of ATP from Ishikawa cells. However, ATP release was not detected after Ishikawa cells were treated with trypsin (Figure 1B).

In addition, cathepsin B (Ctsb), a cysteine proteinase, is expressed in trophoblast cells for the extracellular degradation of the uterine matrix during mouse embryo implantation.^29^ Single‐cell RNA‐Seq data of human preimplantation embryos showed that the expression level of Ctsb mRNA was highest in the blastocyst stage compared with the zygote stage and the 2‐cell, 4‐cell, 8‐cell embryo or morula stage.^30^ When Ishikawa cells were treated with human recombinant Ctsb protein, there was no detectable ATP release (Figure 1C).

Lactate produced by mammalian blastocysts may promote embryo implantation.^31^ The uterine lumen of the mouse is under acidic conditions during the peri‐implantation period.^32^ We hypothesized that embryonic lactate might induce ATP release. When we examined the concentration of lactate in the medium from cultured human embryos, the concentration of lactate was significantly higher than that in the control medium (Figure 1D). This result suggested that human embryos might release lactate during the preimplantation period. When Ishikawa cells were treated with 0, 10, 15 or 20 mmol/L lactate for 1 hour, the highest release of ATP was detected at 20 mmol/L (Figure 1E). Furthermore, after Ishikawa cells were treated with 20 mmol/L lactate from 10 minutes to 3 hours, ATP release was time‐dependently increased, reaching its peak at 1 hour and dropping sharply to basal levels at 3 hours (Figure 1F). Therefore, human blastocyst–derived lactate could stimulate non‐lytic ATP release from epithelial cells.

### 3.3 Lactate promotes non‐lytic ATP release from epithelial cells via connexins

**Figure 2:**
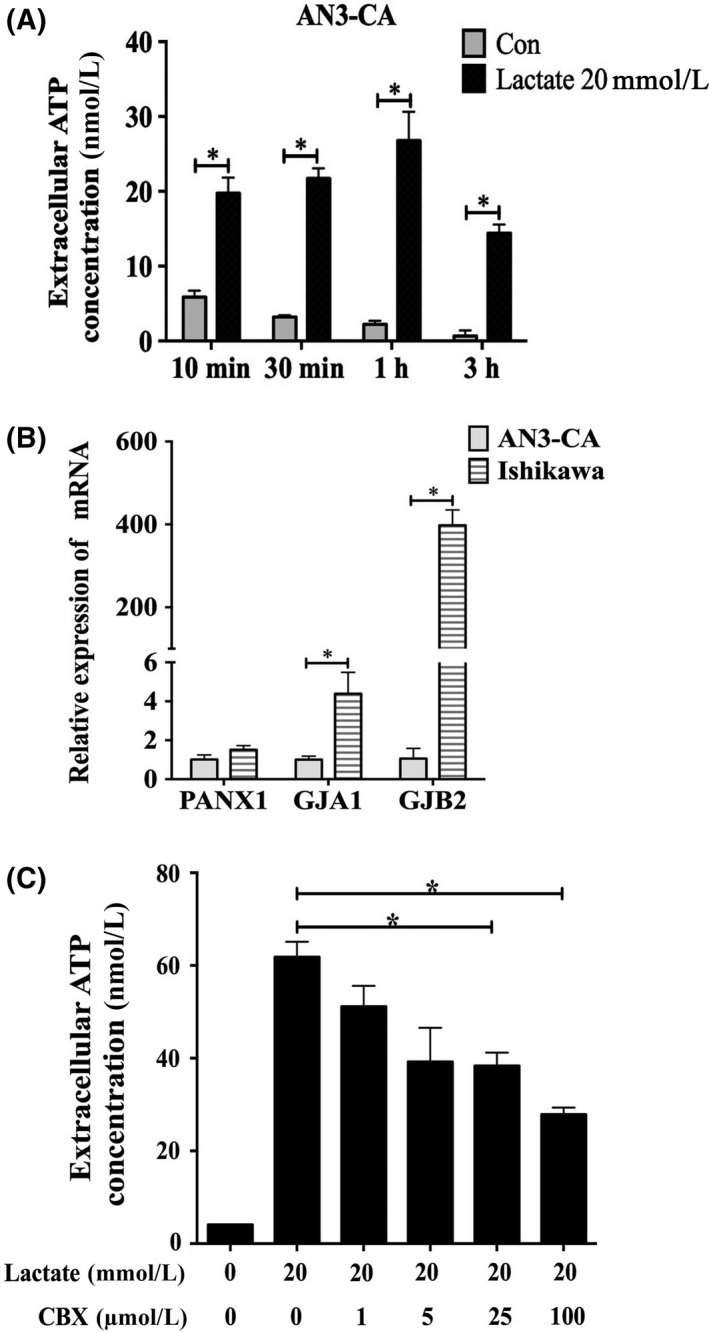
Lactate‐induced ATP release from receptive epithelial cells via connexins. A, Levels of extracellular ATP released when AN3‐CA cells were treated with 20 mmol/L lactate for 10 min, 30 min, 1 h and 3 h, respectively. B, The mRNA expression levels of Panx1, Cx26 (GJB2) and Cx43 (GJA1) were performed by RT‐PCR in two human endometrial epithelial cell lines. C, The effects of different concentrations of CBX on extracellular ATP levels when Ishikawa cells were treated with 20 mmol/L lactate for 1 h. Data are presented as the mean ± SD, *P < .05

We aimed to explore whether lactate‐induced non‐lytic ATP release is related to the expression of hemichannel proteins. When AN3‐CA, another human endometrial epithelial cell line, was treated with 20 mmol/L lactate for 10 minutes, 30 minutes, 1 hour and 3 hours, ATP release was increased in different time points (Figure 2A). Diffusional ATP was released via pannexin and connexin hemichannels in various cell types under physiological conditions.^11^ We examined the expression of pannexins and connexins in Ishikawa and AN3‐CA cells. Surprisingly, the expression levels of Cx26 and Cx43 mRNA (known as GJB2 and GJA1, respectively) were higher in Ishikawa cells than in AN3‐CA cells. However, Panx1 expression was not significantly different between the two cell lines (Figure 2B). Carbenoxolone (CBX), a nonselective gap junction inhibitor, significantly inhibited lactate‐induced ATP release from Ishikawa cells (Figure 2C). Therefore, these results implied that lactate might promote ATP release from human endometrial receptive epithelial cells via the connexin hemichannel.

### 3.4 Extracellular ATP induces IL8 secretion from epithelial cells to promote decidualization

**Figure 3:**
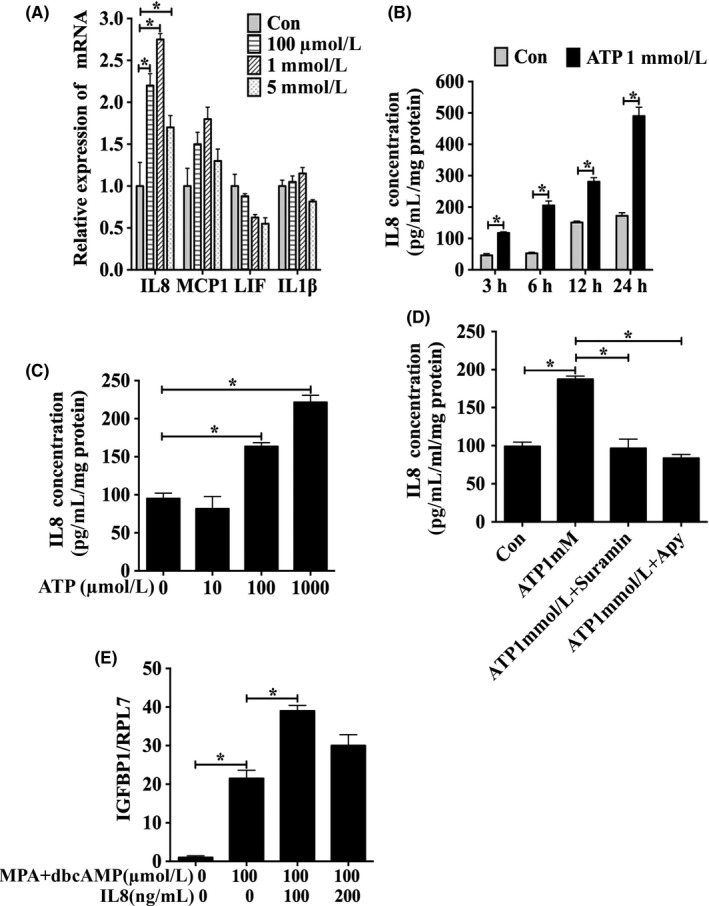
ATP‐induced IL8 secretion from Ishikawa cells. A, Levels of IL8, MCP1, LIF and IL1β mRNA expression when Ishikawa cells were treated by different concentrations of ATP for 1 h. B, Secreted IL8 concentrations were detected by enzyme‐linked immunoassay when Ishikawa cells were treated with 1 mmol/L ATP at different time points. C, Secreted IL8 concentrations when Ishikawa cells were treated with different concentrations of ATP for 24 h. D, Effects of suramin and apyrase treatment for 24 h on ATP‐induced IL8 secretion in Ishikawa cells. E, Effects of exogenous IL8 on IGFBP1 mRNA expression in human endometrial stromal cells under in vitro decidualization for 2 d. Data are presented as the mean ± SD, *P < .05

It has been shown that released ATP from necrotic cells triggers a sterile inflammatory response, resulting in the subsequent release of proinflammatory cytokines.^33^ Therefore, we speculated that extracellular ATP from epithelial cells should act on adjacent epithelial cells to promote the expression of proinflammatory cytokines. When we examined the effects of ATP on the expression of IL8, MCP1, LIF and IL1β, the expression of IL8 mRNA in Ishikawa cells was obviously upregulated by different concentrations of ATP for 1 hour. ATP treatment had no detectable effects on the expression of MCP1, LIF and IL1β (Figure 3A). The secretion of IL8 by Ishikawa cells was significantly increased by 1 mmol/L ATP at 3, 6, 12 and 24 hours, respectively (Figure 3B). Treatments of Ishikawa cells with 100 μmol/L or 1 mmol/L ATP for 24 hours stimulated an increase in IL8 secretion (Figure 3C). ATP‐stimulated IL8 secretion was abrogated by apyrase, an ectonucleotidase that hydrolyses extracellular ATP. Suramin, a nonspecific antagonist of purinergic receptors, also significantly suppressed ATP‐induced IL8 secretion (Figure 3D).

Then, we explored whether IL8 secretion was involved in decidualization. When T‐HESCs were treated with 100 ng/mL IL8 for 2 days under in vitro decidualization, the expression of IGFBP1 mRNA was significantly increased (Figure 3E). Taken together, these results suggested that the IL8 secretion stimulated by extracellular ATP in Ishikawa cells could promote the process of in vitro decidualization of human endometrial stromal cells.

### 3.5 Extracellular ATP induces decidualization

**Figure 4:**
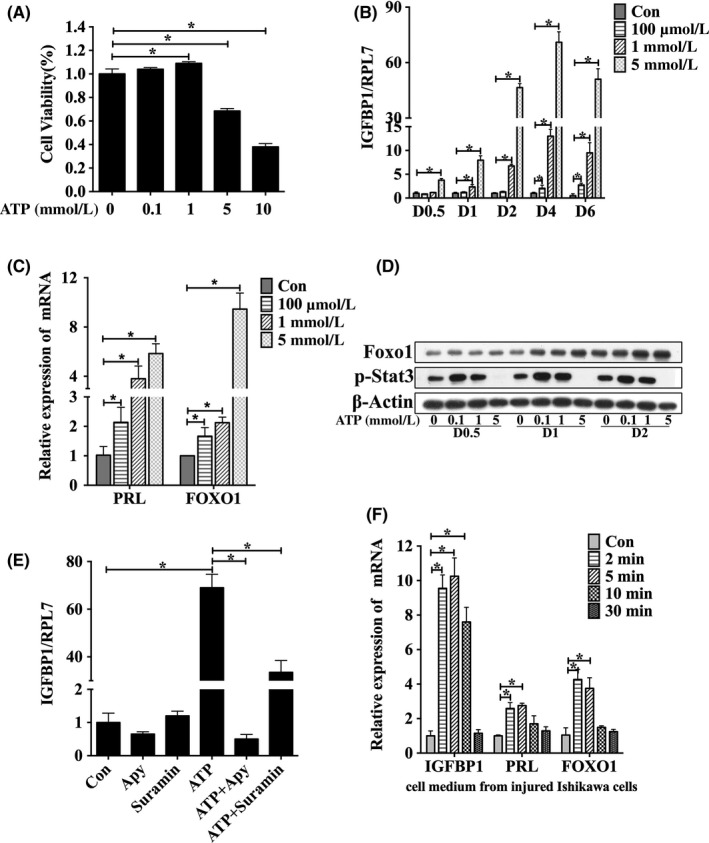
Extracellular ATP induces in vitro decidualization of T‐HESCs. A, Effects of treatment with different concentrations of ATP for 2 d on the viability of T‐HESCs. B, Effects of treatment with different concentrations of ATP on IGFBP1 mRNA expression from days 0.5 to 6. C, Effects of treatment with different concentrations of ATP for 2 d on PRL and FOXO1 mRNA expression. D, Effects of treatment with different concentrations of ATP on the p‐Stat3 and Foxo1 protein levels from days 0.5 to 2. E, Effects of treatment with apyrase and suramin for 2 d on ATP‐induced IGFBP1 mRNA expression. F, Effects of treatment with conditioned medium from scratched Ishikawa cells on in vitro decidualization in T‐HESCs for 2 d. Data are presented as the mean ± SD, *P < .05

Our data suggested that ATP‐induced IL8 secretion by the luminal epithelium promoted in vitro decidualization. However, we wondered whether ATP released from the luminal epithelium could induce in vitro decidualization. The effect of ATP on the viability of T‐HESCs was examined using a CCK8 kit. ATP at 0.1 mmol/L and 1 mmol/L had no detectable effects on viability. However, viability was significantly reduced by 5 mmol/L ATP and 10 mmol/L ATP at 48 hours (Figure 4A). Then, ATP at concentrations from 100 μmol/L to 5 mmol/L were used to examine changes in the expression of IGFBP‐1. Compared with that of the control, the expression levels of IGFBP1 mRNA in the ATP‐treated cells gradually increased in a dosage‐ and time‐dependent manner from days 0.5 to 2, and the increase was especially notable on day 2. However, the levels of IGFBP1 mRNA were similar on days 4 and 6 to those on day 2 (Figure 4B). In addition, the mRNA levels of PRL and FOXO1, which are decidualization markers, were clearly upregulated in T‐HESCs by 100 μmol/L to 5 mmol/L ATP on day 2 (Figure 4C). The protein level of FOXO1 was strongly induced by ATP in a dosage‐ and time‐dependent manner in T‐HESCs. The p‐STAT3 level was increased only by 100 μmol/L and 1 mmol/L ATP at different time points (Figure 4D). The ATP‐induced IGFBP‐1 increase was significantly suppressed by apyrase (a nucleoside triphosphate hydrolase) or suramin (a P2‐purinergic receptors antagonist) (Figure 4E).

Because ATP was released from scratched Ishikawa cells, conditioned medium from scratched Ishikawa cells was used to culture T‐HESCs. Unexpectedly, the cell medium collected from the scratched Ishikawa cells at short time points (2, 5 and 10 min) promoted the expression of IGFBP1, PRL and FOXO1 on day 2. However, the cell medium collected from scratched Ishikawa cells at 30 minutes had no effect (Figure 4F). Therefore, these results indicated that extracellular ATP could indeed promote the expression of decidualization marker genes in T‐HESCs.

**Figure 5:**
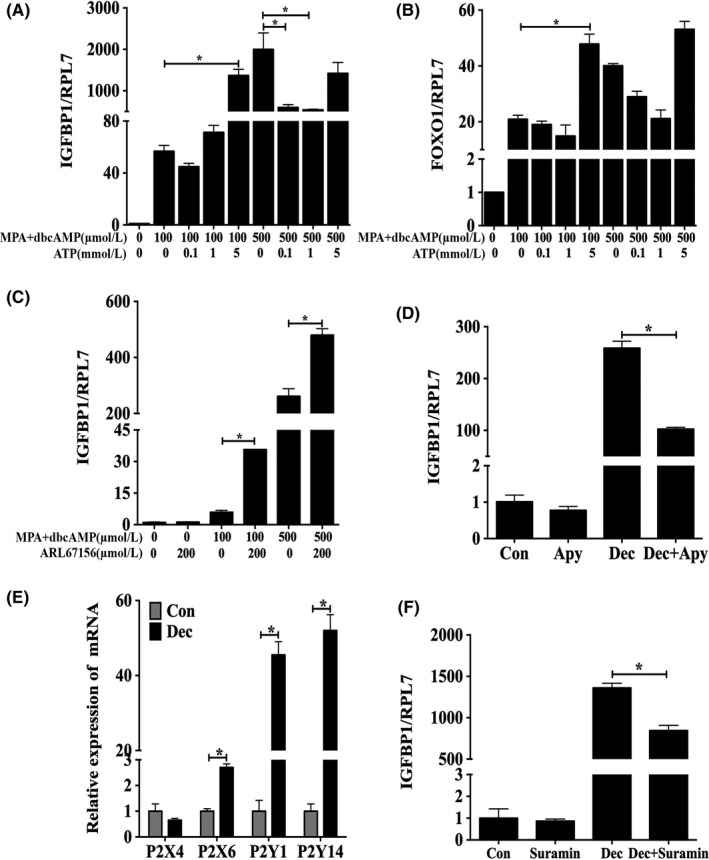
Extracellular ATP for 2 d promotes in vitro decidualization. A, Effects of treatment with different concentrations of ATP on IGFBP1 mRNA expression under in vitro decidualization induced by 100 and 500 μmol/L db‐cAMP. B, Effects of treatment with different concentrations of ATP on Foxo1 mRNA expression under in vitro decidualization induced by 100 and 500 μmol/L db‐cAMP. C, ARL67156 stimulates IGFBP1 mRNA expression under in vitro decidualization induced by 100 and 500 μmol/L db‐cAMP. D, Apyrase inhibits IGFBP1 mRNA expression under in vitro decidualization induced by 500 μmol/L db‐cAMP. E, The mRNA expression levels of P2X4, P2X6, P2Y1 and P2Y14 under in vitro decidualization induced by 500 μmol/L db‐cAMP. F, Suramin inhibits IGFBP1 mRNA expression induced by 500 μmol/L db‐cAMP. Data are presented as the mean ± SD, *P < .05

Because decidualization was induced by ATP, we aimed to explore how ATP regulated decidualization. ATP was stimulatory for IGFBP‐1 and FOXO1 expression when in vitro decidualization was induced by 100 μmol/L db‐cAMP and MPA for 2 days. However, ATP had an inhibiting effect when 500 μmol/L db‐cAMP and MPA were used (Figure 5A,5B). ATP‐induced IGFBP‐1 expression was further enhanced by ARL67156, an ecto‐ATPase inhibitor for preventing ATP degradation (Figure 5C). Apyrase significantly downregulated ATP‐stimulated IGFBP‐1 expression (Figure 5D).

Compared with those of the control, the mRNA expression levels of p2x6, p2y1 and p2y14, which are P2‐purinergic receptors of extracellular ATP receptors, were upregulated under in vitro decidualization for 2 days, with the mRNA expression levels of p2y1 and p2y14 being higher than those of p2x6 (Figure 5E). Suramin, a broad spectrum antagonist of P2Y purinergic receptors, was able to abrogate ATP‐induced expression of IGFBP1 (Figure 5F). These results showed that extracellular ATP might enhance the process of decidualization of T‐HESCs via its P2Y receptors.

## 4 DISCUSSION

Our data indicated that embryo‐derived lactate might act as an inducer of ATP release from human endometrial receptive epithelial cells to regulate the decidualization of stromal cells via IL8. There is an increase in lactate production by human blastocysts under in vitro culture,^34^ and the increase in glucose uptake during the blastocyst period may be accounted for by the appearance of lactate in the incubation medium.^35^, ^36^ Lactate produced by mammalian blastocysts should provide a favourable microenvironment for embryo implantation and invasion.^31^ In our study, there was a significant increase in lactate production in human embryo‐conditioned medium compared with the control medium. In mice, the uterine luminal epithelium is acidized during embryo implantation.^32^ It is possible that blastocyst‐derived lactate leads to local acidification of the uterine epithelium during human embryo implantation. In cultured mouse cortical neurons, lactate‐induced ATP is released through pannexins.^37^ Lactate provides a weakly acidic environment and Cx26 hemichannel has a flexibly opening pore allowing unimpeded passage of ions under the acidic condition.^38^ Multiple mechanisms have been proposed to regulate ATP release through connexin (Cx) hemichannels.^39^ There is an increase in connexin 26 in human endometrial glandular and luminal epithelial cells during the mid‐secretory phase of the menstrual cycle.^40^ Human in vitro decidualization of endometrial stromal cells can be blunted by gap junction blockade.^41^ In rodents, Cx26 is locally expressed in uterine epithelial cells at implantation sites.^42^ We also found that the expression of Cx26 mRNA was significantly higher and connexins inhibitor reduces lactate‐induced ATP release from intracellular to extracellular in human endometrial receptive epithelial cells, indicating that lactate‐induced ATP release from receptive epithelial cells may be mediated by Cx26.

In humans and rodents, embryo implantation relies on a proinflammatory response induced by several proinflammatory factors, including PGE_2_, TNF, IL6, IL1β and IL8.^20^ Extracellular ATP may act as danger molecule in the testicular environment and able to promote sterile inflammatory responses by upregulating IL6 and IL1β.^43^ IL8 production and secretion are induced by ATP in human oesophageal epithelial cells and glioma cells.^44^, ^45^ Our data demonstrated that ATP‐stimulated IL8 from epithelial cells regulates the decidualization of stromal cells. In the human endometrium, IL8 is located in the surface epithelium and glands throughout the menstrual cycle.^46^ Human blastocysts can upregulate IL8 mRNA expression and protein production in endometrial epithelial cells.^47^ Epithelial and stromal staining for CXCR1 (IL8 receptor) reaches a peak at the mid‐secretory phase.^48^ IL‐8 knockdown also compromises the process of in vitro decidualization.^49^ IL8 has been shown to promote human trophoblast invasion by increasing the activity of matrix metalloproteinase (MMP2, MMP9) in in vitro models and contribute to the uterine natural killer (uNK) cell stimulation of trophoblast invasion.^50^ Clearance of senescent decidual cells by uNK cells involves the secretion of IL8 in cycling human endometrium.^51^ Based on our data, ATP‐stimulated IL‐8 secretion from receptive epithelial cells should be important for embryo invasion and stromal cell decidualization.

In addition, our results showed that extracellular ATP can directly induce the decidualization of T‐HESCs in vitro. Suramin is a broad spectrum antagonist for P2Y purinergic receptors.^52^ We also found that suramin can suppress the process of decidualization in vitro, suggesting that the expression of purinergic receptors is important for ATP signal transduction in vitro decidualization of human endometrial stromal cells. Extracellular ATP activates the phosphorylation of ERK1/2 leading to the induction of matrix metalloproteinase expression in human endometrial stromal cells.^53^ Further research showed that ATP induces the translocation of ERK1/2 into the nucleus and inhibits the cell viability via P2Y2 receptor in human endometrial stromal cells.^54^ P2Y2 receptor is involved in the activation of p‐Stat3 in the process of ATP‐stimulated regeneration of primary sensory axons,^55^ whereas the activation of ERK1/2 and Stat3 are required for successful decidualization in human endometrial stromal cells and Stat3 is known as a downstream target of ERK1/2.^56^ Therefore, we speculated that ATP enhances human decidualization in vitro via P2Y2/p‐ERK/p‐Stat3 signalling pathway.

Local endometrial injury (also known as endometrial “scratching”) is factitious damage to the endometrium, usually induced by a Pipelle catheter.^57^ Although there is a discrepancy in clinical data, many randomized and nonrandomized controlled studies have demonstrated that endometrial injury should be beneficial for embryo implantation in women with repeated implantation failure.^58^ Local injury of the endometrium induces an inflammatory response associated with increased macrophages/dendritic cells.^59^ Intentional endometrial injury may increase implantation potential by enhancing endometrial angiogenesis in mice.^60^ In our study, abundant ATP was released from damaged human endometrial epithelial cells. The concentration of extracellular ATP is controlled by specific ectonucleotidases.^61^ In cyclic human endometria, ENTPD1 (CD39) is strongly expressed in the endothelial cells of stromal blood vessels and in stromal cells. NT5E (CD73) is located in the glandular epithelium and stroma.^62^ Our results also demonstrated that ATP from damaged Ishikawa cells was rapidly hydrolysed after 5 minutes. This may be related to the expression of ectonucleotidases in the human endometrium. In our study, inhibition of ectonucleotidase activity also promoted the decidualization of T‐HESCs. In mice, artificial decidualization can be induced by either mechanical injury or sesame oil injection in the pseudopregnant uterine lumen.^63^ Therefore, it is plausible to speculate that ATP release from endometrial injury may optimize decidualization in women with repeated implantation failures.

**Figure 6:**
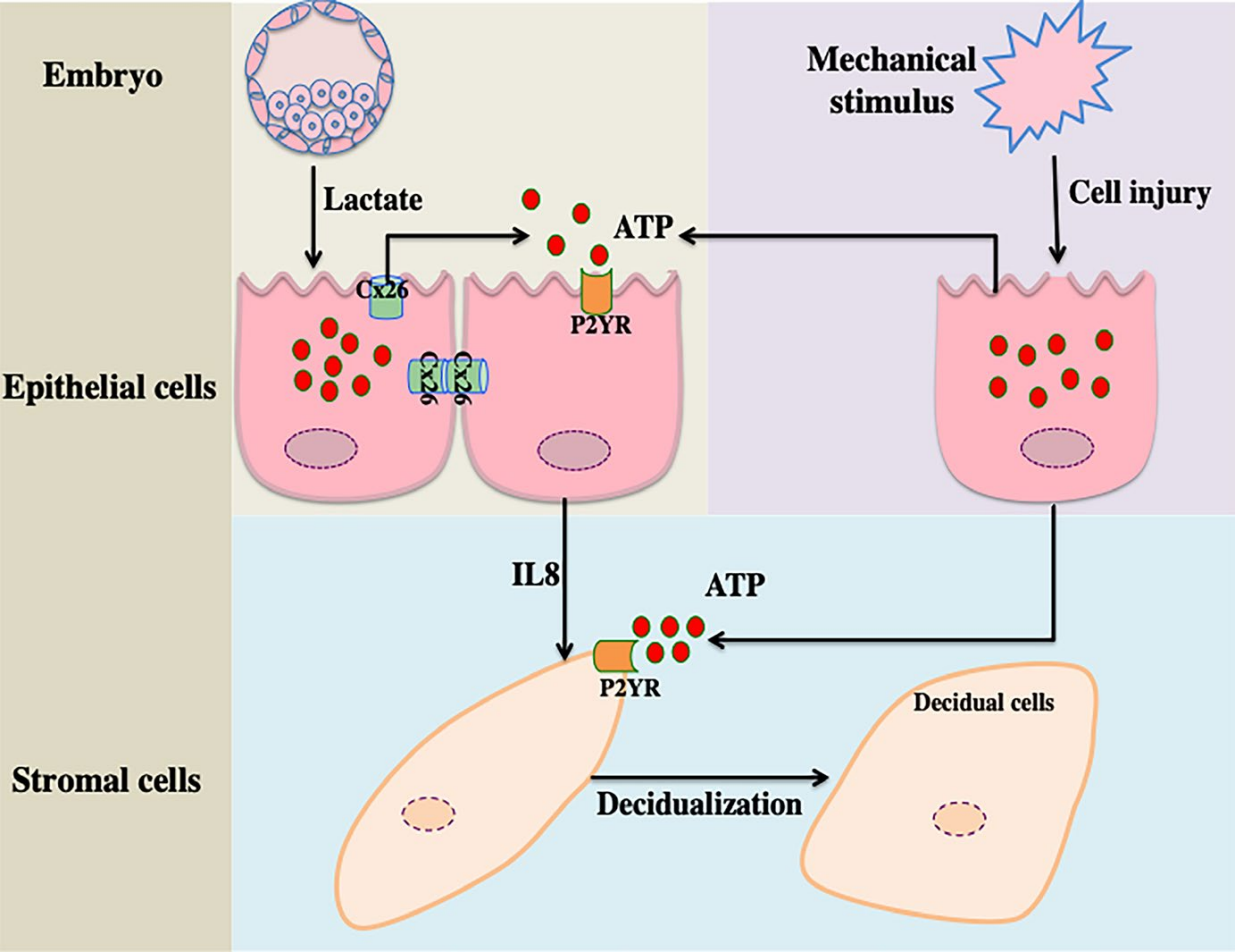
The model of ATP action in the human blastocyst‐endometrial interaction. Released ATP from injured endometrial epithelial cells directly induces the decidualization of stromal cells via P2YRs. In addition, embryo‐secreted lactate may stimulate ATP release from receptive epithelial cells via Cx26. ATP induces IL8 secretion from adjacent epithelial cells to promote the decidualization of stromal cells

Take together, our results showed that ATP released from uterine epithelial cells regulates the decidualization of stromal cells through IL‐8. Injured epithelial cells release ATP to directly promote decidualization. These data should shed light on understanding the interaction between blastocysts and the receptive endometrium (Figure 6).

## CONFLICT OF INTEREST

I certify that neither my co‐authors nor I have a conflict of interest as described above that is relevant to the subject matter or materials included in this work.

## AUTHOR CONTRIBUTIONS

XWG and ZMY designed experiments in this study; YY, TL, ZCC, JMP and XWG performed research and acquired data; YY, XWG, JPO and ZMY analysed data; TL, TF, XWG and ZMY interpreted data; XWG, JPO and ZMY wrote the paper and revised it; and all authors gave the final approve of the version of to be published.

## Data Availability

The data that support the findings of this study are available from the corresponding author upon reasonable request.
